# Spontaneous Facial Actions Map onto Emotional Experiences in a Non-social Context: Toward a Component-Based Approach

**DOI:** 10.3389/fpsyg.2017.00633

**Published:** 2017-05-04

**Authors:** Shushi Namba, Russell S. Kabir, Makoto Miyatani, Takashi Nakao

**Affiliations:** ^1^Graduate School of Education, Hiroshima UniversityHiroshima, Japan; ^2^Department of Psychology, Hiroshima UniversityHiroshima, Japan

**Keywords:** emotions, facial expressions, spontaneous, components, non-social

## Abstract

While numerous studies have examined the relationships between facial actions and emotions, they have yet to account for the ways that specific spontaneous facial expressions map onto emotional experiences induced without expressive intent. Moreover, previous studies emphasized that a fine-grained investigation of facial components could establish the coherence of facial actions with actual internal states. Therefore, this study aimed to accumulate evidence for the correspondence between spontaneous facial components and emotional experiences. We reinvestigated data from previous research which secretly recorded spontaneous facial expressions of Japanese participants as they watched film clips designed to evoke four different target emotions: surprise, amusement, disgust, and sadness. The participants rated their emotional experiences via a self-reported questionnaire of 16 emotions. These spontaneous facial expressions were coded using the Facial Action Coding System, the gold standard for classifying visible facial movements. We corroborated each facial action that was present in the emotional experiences by applying stepwise regression models. The results found that spontaneous facial components occurred in ways that cohere to their evolutionary functions based on the rating values of emotional experiences (e.g., the inner brow raiser might be involved in the evaluation of novelty). This study provided new empirical evidence for the correspondence between each spontaneous facial component and first-person internal states of emotion as reported by the expresser.

## Introduction

Imagine all the ways that your face or the faces of those you care about have been a shorthand for the precious and teachable moments of your life. The expressions we make and the emotions they entail are important because our interpretations of them have personal and social consequences. Facial expressions covary with internal states of the mind, such as emotions, appraisals, and intentions (Ekman, 1994; McLellan et al., 2010; Gentsch et al., 2015). Successful interpersonal communication relies on the ability to convey and interpret emotions accurately, and in this manner, understanding facial expressions is vital for regulating our interactions with others.

Several studies have established the coherence between facial expressions and emotion by observing and measuring facial activity (Ekman et al., 1980; Mauss et al., 2005), facial recognition (Ekman and Friesen, 1971; Horstmann, 2003), facial electromyography (EMG, Cacioppo et al., 1986, 1992) and facial feedback (McIntosh, 1996; Soussignan, 2002). In experimental studies, Mauss et al. (2005) indicated that there was an association between observer ratings of facial behavior elicited by films generating amusement or sadness and the expressers’ continuous self-report of emotional experience. Levenson et al. (1992) also confirmed that voluntarily making facial patterns for specific emotions like anger, fear, sadness, disgust, and happiness generates specific emotional experiences and autonomic nervous system activity, irrespective of culture. In this way, relationships between the face and emotions are known to exist and are considered to be universal (e.g., Matsumoto et al., 2008).

Most prior research on the correspondence between facial expressions and emotion has been conducted on the grounds of Basic Emotion Theory (BET: Ekman, 1994, 2003). BET hypothesized that basic emotions such as happiness, anger, disgust, fear, sadness, and surprise have an individual and prototypical facial pattern (Tomkins, 1962; Ekman et al., 2002). For example, facial expressions of happiness are comprised of the contraction of the *zygomatic major* muscle and the *orbicularis oculi* muscle. Many staple psychological studies (e.g., Whalen et al., 1998; Balconi and Canavesio, 2016) have implemented facial stimuli (Pictures of Facial Affect; Ekman and Friesen, 1976) that rely on this theory.

However, there are two main criticisms of the assumptions that BET makes about facial expressions. One is that the complete prototypical facial patterns described by BET have rarely been observed in empirical studies. Fernández-dols and Crivelli (2015) pointed out that these prototypical facial patterns were merely described in the literature according to Darwin’s (1872) intuitions, which were then reinterpreted by Allport (1924) and later by Tomkins (1982), rather than arrived at by empirically testing the typical facial expressions that appear during the experience of intense emotions. Thus, prototypical facial expressions might differ from those that might be expressed when one actually experiences emotions. Indeed, Fernández-dols et al. (1997) showed that spontaneous facial expressions elicited by emotion elicitation films do not accord with the prototypical facial patterns stipulated by BET. Reisenzein et al. (2013) also reported that the relationships between specific emotions and prototypical facial expressions characterized by BET are very weak because the majority of experimental evidence has not observed these prototypical facial patterns when people experience the emotions to which they are meant to correspond. One specific example is the Duchenne smile which is characterized by the shrinkage of the oculi muscles (Ekman, 1992). Although previous studies based on BET have considered this smile the signature marker for facial expressions of experienced happiness (Frank et al., 1993), recent studies have demonstrated that Duchenne smiles can be feigned (Krumhuber and Manstead, 2009; Namba et al., 2016), and non-Duchenne smiles have also been shown to occur in experiences of happiness (Krumhuber et al., 2014). Therefore, it is necessary to accumulate data for facial expressions in conditions where participants are actually experiencing emotions.

The other criticism is that BET oversimplifies the link between a given full facial expression and a single emotion. Recent studies have advocated the importance of considering each facial action as a separate component rather than a one-to-one holistic correspondence because the component-centered assumption more comprehensively covers the wide variety of facial patterns that are known to occur in our everyday lives (e.g., Krumhuber and Scherer, 2011; With and Kaiser, 2011; Scherer et al., 2013; Korb et al., 2014). Smith and Scott (1997) touted the advantages of a component-based approach as it allows for individual components to be fine-tuned to explain entire facial expressions from the bottom up. In research on the facial feedback hypothesis, Lewis (2012) found that the voluntary action of each facial component like the brow lowerer might elicit a change of internal states within the expresser.

Taken together, it is necessary to highlight the role of each facial component when considering which factors of emotion cause facial expressions. There are few empirical investigations of spontaneous facial components when people experience emotions.

While several studies have investigated specific facial actions that are expressed naturally when a person actually experiences a number of emotions (e.g., Galati et al., 2003; Matsumoto and Willingham, 2009; Mavadati et al., 2013; Zhang et al., 2014), these findings included two important shortcomings. One pervasive challenge has been identifying the precise facial actions that correspond to emotional experiences. Several studies employed facial electromyography as it provides sensitive detection of subtle facial actions in areas like the brow, cheek, and mouth regions (Lang et al., 1993; Dimberg et al., 2000). However, when investigating naturally occurring spontaneous facial expressions, the attachment of electrodes to the face required for EMG measurement creates an unnatural setting that may potentially disturb the natural occurrence of facial expressions (Ekman et al., 2002). Murata et al. (2016) showed that EMG methods can be problematic for detecting facial actions related to emotion because the necessary placement of electrodes on key areas of the face pick up all muscle tensions indiscriminately in real-time. Murata et al. (2016) also mentioned that if the number of trials is low, as frequently occurs in spontaneous facial expression studies, EMG activity can be prone to noise in facial detection. Thus, a less invasive and more discernible approach is desirable to measure and analyze naturally occurring spontaneous facial expressions.

The other arguably more important shortcoming, however, has been the lack of experimental control for the modifying effects of expressive intent inherent to the display of facial expressions in public settings. Many studies obtained data for spontaneous facial expressions of emotion in an experimental environment where participants were in the presence of others (Galati et al., 2003; Matsumoto and Willingham, 2006) or otherwise conscious of being recorded (Mavadati et al., 2013; Zhang et al., 2014). Thus, previously reported facial expressions may reflect the obstructive factors of social modification like intention in addition to their emotional content (Chovil, 1991; Kunzmann et al., 2005; Gosselin et al., 2010, 2011). Furthermore, Gosselin et al. (2010) found that people can deliberately manipulate prototypical facial patterns of each emotion with expressive and conscious intent. In order to address these gaps in the literature for spontaneous facial expressions, it is necessary to accumulate evidence for observable facial components related to first-person accounts of emotional experiences in the absence of the social factors that may beget expressive intent.

To elucidate new relationships between facial expressions and emotions beyond the prototypical facial pattern characterized by BET, we sought to investigate each observable facial component while controlling for the plausible obstruction of expressive intentions generated in the presence of others. We opted to use data of secretly recorded facial behaviors from participants as they watched emotion elicitation films in a room by themselves. We subsequently analyzed each facial component by corroborating the peak facial expressions that participants elicited from watching film clips with self-reports of their actual emotional experiences taken upon watching them.

## Materials and Methods

We reinvestigated the spontaneous facial actions and emotional experiences reported in Namba et al. (2016) for potential relationships. Namba et al. (2016) recorded spontaneous and posed facial expressions to compare their morphological and dynamic content, but the current study was explicitly focused on creating a predictive model that corroborates spontaneous facial components with emotional experiences. We applied regression models to derive relationships for each facial component and modeled the occurrences of each component using the rating values of emotional experiences as predictor variables, and vice versa.

### Participants

Data were collected from 31 undergraduate students (13 male; *M*_age_ = 20.19, *SD* = 1.37, range = 18–24) at Hiroshima University. They participated on a voluntary basis and were given a monetary compensation of 500 yen after the experiment. All participants were native Japanese speakers with normal or corrected-to-normal vision. There was no evidence of the presence of a neurological or psychiatric disorder. Written informed consent was obtained from each participant before the investigation, in line with a protocol approved by the Ethical Committee of the Graduate School of Education, Hiroshima University.

### Emotion Elicitation

We used four film clips that were designed to elicit amusement, disgust, sadness, and surprise, respectively: *When Harry Met Sally, Pink Flamingos, The Champ*, and *Capricorn*. These films were standardized by Gross and Levenson (1995). Sato et al. (2007) confirmed these four films as eliciting the desired emotional experiences in Japanese participants. After the first 10 participants were collected, we noticed that *When Harry Met Sally* elicited unintended negative emotions such as embarrassment and confusion more than the target emotion of amusement. Therefore, we switched to another one, *Torololo Cat*, which was subsequently labeled amusement 2. The clip lengths were as follows: 155 s for amusement 1 (*When Harry Met Sally*), 51 s for amusement 2 (*Torololo Cat*), 30 s for disgust (*Pink Flamingos*), 171 s for sadness (*The Champ*), and 49 s for surprise (*Capricorn*). All the film clips had sound. As participants watched these film clips, we recorded their facial expressions secretly during each session.

For the emotional assessment of each film, we used the following two methods akin to previous studies (Gross and Levenson, 1995; Sato et al., 2007). The first was a 16-item self-report inventory for discrete emotional assessment. The items included amusement, anger, arousal, confusion, contempt, contentment, disgust, embarrassment, fear, happiness, interest, pain, relief, sadness, surprise, and tension on a 9-point scale, ranging from 0 (*not at all*) to 8 (*the strongest in my life*). The order of the 16 items was randomized for each participant. The other assessment was the affect grid developed by Russell et al. (1989). The affect grid estimates emotional state in terms of valence ranging from 1 (*unpleasant*) to 9 (*pleasant*), and arousal ranging from 1 (*sleepiness*) to 9 (*high arousal*).

### Procedure

We wanted to suppress the influence of social aspects to the greatest extent possible. Therefore, the experiments were performed individually. Participants received instructions to watch the films as they were presented on the PC screen (VPCF14AFJ, SONY), and to evaluate the emotions elicited by each film after viewing it. Also, participants were given inter-trial intervals ranging between 25 and 35 s while being instructed to clear their mind of all thoughts. The order of the films was counterbalanced using a Latin Square design. Participants assessed their emotional state using the 16-item self-report inventory of discrete emotions and the affect grid. These two scales were also counterbalanced. While they were watching the films, we secretly recorded their facial expressions using the camera embedded on the PC screen.

During debriefing sessions, the participants were informed of the previously undisclosed recording of their facial expressions via the embedded camera. They were then given the option to sign a second consent form permitting us to use their recorded facial expressions for analysis or to have us delete the data. If consent was not obtained, then we deleted the recorded data in front of the participant.

### Facial Data

In the debriefing session, one woman refused to permit the use of her facial expressions. The spontaneous facial expressions of another participant were also not available because of problems with the embedded camera. Consequently, 29 spontaneous facial expressions for each emotion totaling 116 facial data were available for the following analyses. The perceptual apex for each facial expression was selected in order to create a model of facial components predicted by emotional rating values.

### Selection of Apex

To detect the apex of each spontaneous facial expression, two evaluators separate from and excluding the experimenter evaluated the recorded facial expressions without sound to not affect their evaluations. They were blinded to the hypothesis of the study. The evaluators watched all 116 videos of spontaneous facial expressions and determined the apex as the frame that seemed to have showed the most morphologically explicit reaction to the emotion elicitation film. The clip lengths of the collected recordings watched by evaluators was consistent with the emotion elicitation films used as the standardized stimulus. For example, the facial expressions of surprise presented to evaluators were those that were elicited by participants and continued for 49 s, in line with the length of the surprise elicitation film (*Capricorn*). The spontaneous facial expressions were presented at a rate of 30 fps. The order of videos was randomized. These apex points can be described as the perceptual apex because they were selected per the judgment of the evaluators.

### Facial Coding

We coded the recorded facial expressions using the Facial Action Coding System (FACS: Ekman and Friesen, 1978) which can objectively describe visible facial movements based on anatomy. FACS defines each facial component as an Action Unit (AU). All apexes for the target expression that were exhibited while watching the emotion elicitation films were coded. A certified FACS coder who passed the FACS final test scored all the apex points for the facial expressions. We excluded blinking (AU45), yawning, and the units that incorporate horizontal or vertical orientation in head movement and gaze direction (AUs 50–66) because our study was explicitly focused on emotional facial movements.

To ensure reliability, a second trained FACS coder scored the same facial data. Agreement regarding the presence of AUs was sufficiently high to indicate intercoder reliability (Agreement = 85.5%; Cohen’s κ = 0.84, *p* < 0.001). We calculated these indexes using R (3.3.1, R Core Team, 2016) and irr packages (Gamer et al., 2012).

### Statistical Analysis

Our goal was to clarify specific facial components when people have actual emotional experiences. We applied logistic regression and multiple regression models to the facial components and emotional rating values. Twenty kinds of AUs were observed in this study: inner brow raiser as AU 1, outer brow raiser as AU 2, brow lowerer as AU 4, upper lid raiser as AU 5, cheek raiser as AU 6, lid tightener as AU 7, nose wrinkler as AU 9, upper lip raiser as AU 10, nasolabial deepener as AU 11, lip corner puller as AU 12, sharp lip puller as AU 13, dimpler as AU 14, lip corner depressor as AU 15, lower lip depressor as AU 16, chin raiser as AU 17, neck tightener as AU 21, lip tightener as AU 23, lips part as AU 25, jaw drop as AU 26, and mouth stretch as AU 27. However, we considered the parts of AUs that did not occur more than 5% as rare events, and removed them from the pool of analysis. As a result, 12 facial actions were regarded as available variables (**Table 1**): AU 1, 2, 4, 5, 6, 7, 10, 12, 14, 23, 25, and 26.

**Table 1:** List of the 12 AUs that emerged in spontaneous facial data collected in a non-social context and their frequencies.

AU	Frequencies	
	*n*	%
AU1: Inner brow raiser	12	10
AU2: Outer brow raiser	9	8
AU4: Brow lowerer	17	15
AU5: Upper lid raiser	12	10
AU6: Cheek raiser	31	27
AU7: Lid tightener	25	22
AU10: Upper lid raiser	16	14
AU12: Lip corner puller	24	21
AU14: Dimpler	9	8
AU23: Lip tightener	8	7
AU25: Lips part	36	31
AU26: Jaw drop	12	10

We investigated two regression models: one where the occurrences of each AU were predicted by all 16 discrete emotional assessments, and another where each emotional experience was predicted by all 12 facial actions. By examining these two bidirectional models, we aimed to accumulate evidence about the relationships between spontaneous facial components and their correspondence to emotional experiences. For clarity, we have not reported the results of the affect grid (available upon request). Simply performing regression models with such a large number of predictors makes it more likely that the results will fail to converge. To stabilize estimation, we decided to forego unnecessary predictors for each model in favor of the stepwise model selection method stipulated by AIC (Venables and Ripley, 2002). The first full model is a logistic regression described as follows:

(1) $$\text{AU}\;=\;\text{inverse}\_\text{logit}\;(\beta_{\text{intercept}}\;\text{+}\;\beta_{\text{amusement}}{\;}^{\text{*}}\;\text{amusement}\;+...+\;\beta_{\text{tension}}{\;}^{*}\;\text{tension})$$

The second full model is a regression described in the following expression:

(2) $$\text{Emotional}\;\text{experience}\;=\beta_{\text{intercept}}\;+\;\beta_{\text{AU1}}{\;}^{*}\;\text{AU1}\;+...+\;\beta_{\text{AU26}}{\;}^{*}\;\text{AU26}$$

Furthermore, we applied the Bonferroni correction to treat the number of outcome variables as a family of hypotheses. The logistic model was set to the Bonferroni adjusted alpha levels of 0.0042 per test (0.05/12). The other model was set to the Bonferroni adjusted alpha levels of 0.0031 per test (0.05/16). In the following results, we used R (3.3.1, R Core Team, 2016), lme4 packages (Bates et al., 2014) and MASS packages (Venables and Ripley, 2002). Only models that showed significant differences in the predictor were reported to avoid unnecessary complexity and improve clarity.

## Results

### Which Emotional Experiences Predicted Each AU?

**FIGURE 1:**
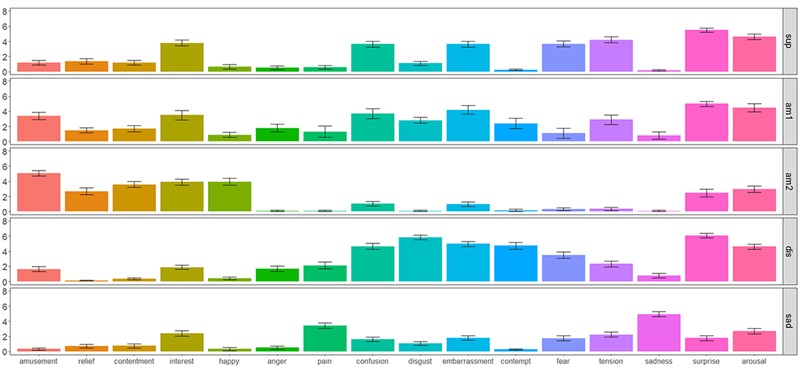
**Mean emotion ratings as a function of the type of eliciting stimulus.** Sup stands for surprise elicitation film (*n* = 29). Am1 stands for amusement 1 elicitation film (*n* = 10). Am2 refers to the amusement 2 elicitation film (*n* = 19). Dis stands for disgust elicitation film (*n* = 29). Sad refers to the sadness elicitation film (*n* = 29).

**FIGURE 2:**
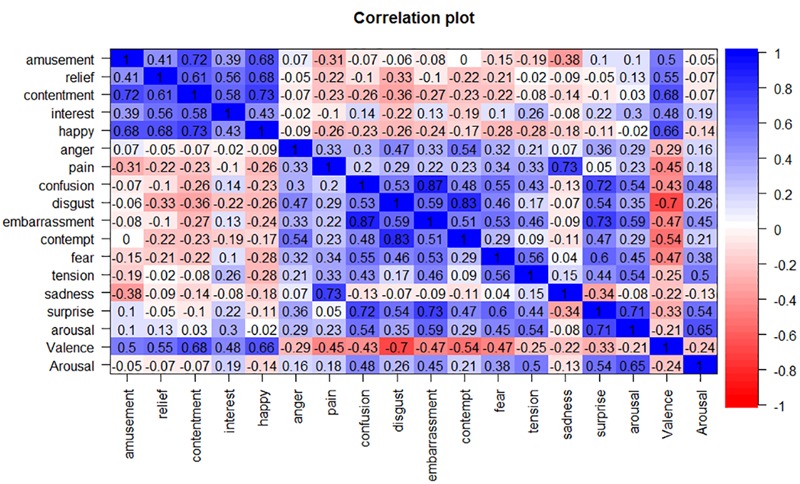
**Correlation matrix of the self-reported emotional rating values.** Blue cells represent the positive relationships between emotional rating values, and red cells represent negative relationships.

**Figure 1** depicts the rating values of emotional experiences for each stimulus to confirm which emotional experiences were elicited in the current study. The matrix table in **Figure 2** represents the correlations among all emotional experiences.

**FIGURE 3:**
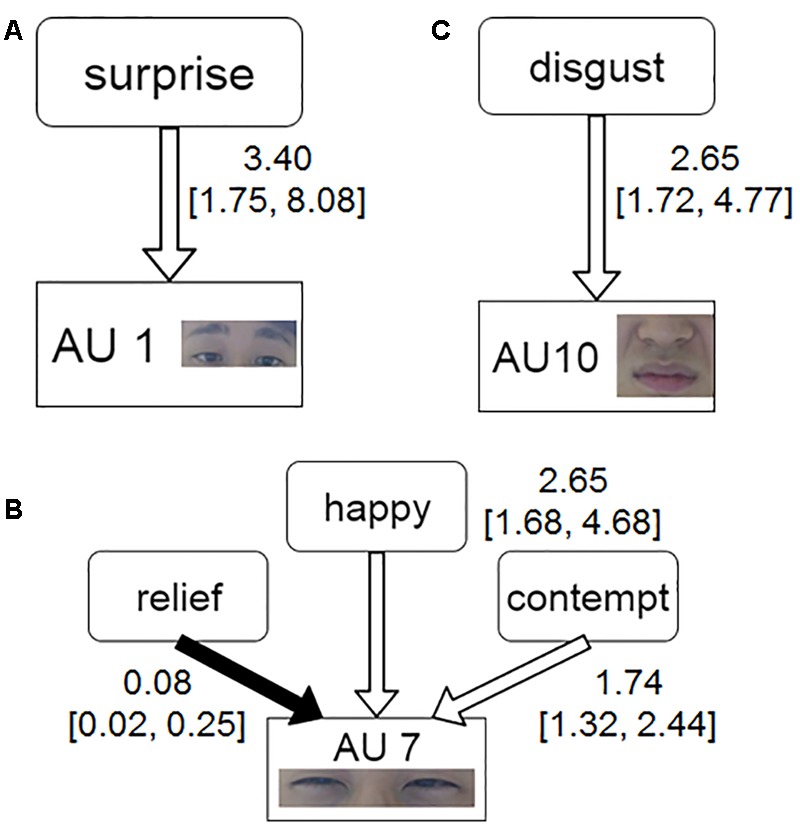
**Model of the occurrence of each AU present in the facial expressions predicted by 16 emotional ratings.** The part labels show emotional experiences predicting the following action units: Action Unit 1 **(A)**, Action Unit 7 **(B)**, and Action Unit 10 **(C)**. The white and black arrows indicate positive and negative relationships, respectively. The coefficients on the side of the node represent the Odds Ratio (OR). The numbers in brackets represent the 95% confidence interval of the OR.

We conducted stepwise logistic regressions (see Eq. 1) to determine if available emotional rating values could explain the dichotomous outcome of whether each AU occurred or not. We applied this analysis to the twenty AUs that could be observed in this study. In all AUs that were observed, three models had significant results: the inner brow raiser (AU 1), the lid tightener (AU 7), and the upper lip raiser (AU 10). **Figure 3** represents the summarized results of the three models and their facial components. The corresponding coefficients stand for the odds ratios (OR), followed by 95% confidence intervals (95% CI). For the inner brow raiser (AU 1), the surprise rating values significantly predicted the occurrence of the inner brow raiser (odds ratio = 3.40 [1.75, 8.08], *p* = 0.02). For the lid tightener (AU 7), the results indicated that the relief, happiness, and contempt rating values significantly predicted the occurrence of the lid tightener (odds ratios = 0.08 [0.02, 0.25]; 2.65 [1.68, 4.68]; 1.74 [1.32, 2.44] respectively, all *p*s < 0.05). The result for the upper lip raiser (AU 10) indicated that the disgust rating values significantly predicted its occurrence (odds ratio = 2.65 [1.72, 4.77] *p* = 0.002).

### Which Facial Actions Predicted Each Emotional Experience?

**FIGURE 4:**
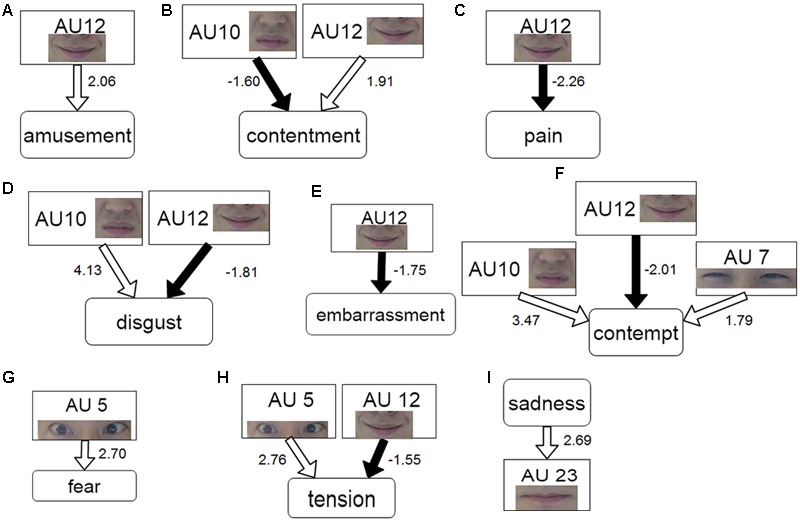
**Model of the emotion ratings predicted by the occurrence of individual AUs.** The part labels show action units predicting the following emotional experiences: amusement **(A)**, contentment **(B)**, pain **(C)**, disgust **(D)**, embarrassment **(E)**, contempt **(F)**, fear **(G)**, tension **(H)**, and sadness **(I)**. The white and black arrows indicate positive and negative relationships, respectively. The coefficients on the side of the node represent significant β.

We also investigated the case of these relationships occurring in the opposite direction, namely in a model where each emotional experience was predicted by the occurrences of several AUs (see Eq. 2). Nine models had significant results: amusement, contentment, pain, disgust, embarrassment, contempt, fear, tension, and sadness. **Figure 4** represents these models. For amusement, the regression result indicated that the occurrences of the lip corner puller (AU 12) significantly predicted the rating values of amusement (β = 2.06, *t* = 3.70, *p* < 0.01). For contentment, the occurrence of the upper lip raiser (AU 10) and the lip corner puller (AU 12) significantly predicted the rating values of contentment (βs = -1.60, 1.91, respectively, *t*s > 3.80, *p*s < 0.005). The pain rating values were significantly predicted by the occurrences of lip corner puller (β = -2.26, *t* = 3.72, *p* < 0.005). As for disgust, the results indicated that the occurrences of the upper lip raiser (AU 10) and the lip corner puller (AU 12) significantly predicted the rating values of disgust (βs = 4.13, -1.81, respectively, *t*s > 3.71, *p*s < 0.01). The model for embarrassment indicated that the occurrence of the lip corner puller (AU 12) was significant (β = -1.75, *t* = 3.47, *p* < 0.05). For contempt, the results indicated that the occurrences of the lid tightener (AU 7), the upper lip raiser (AU 10) and the lip corner puller (AU 12) significantly predicted the rating values of contempt (βs = 1.79, 3.47, and -2.01, respectively, *t*s > 3.95, *p*s < 0.005). As for fear, the occurrences of the upper lid raiser (AU 5) significantly predicted the rating values of fear (β = 2.70, *t* = 3.16, *p* < 0.05). For tension, the occurrences of the upper lid raiser (AU 5) and the lip corner puller (AU 12) were significant predictors (βs = 2.76, -1.55, respectively, *t*s > 3.28, *p*s < 0.05). Finally, the model of sadness indicated that the occurrence of the lip tightener (AU 23) was the significant predictor (β = 2.69, *t* = 3.44, *p* < 0.05).

## Discussion

This study modeled the occurrences of each AU from peak facial expressions in emotional experiences that were then set as predictors, and vice versa, to test the assumptions put forth by BET. Although these results showed that some of the facial components mapped across actual emotional experiences in keeping with BET, we found that the relative importance of each facial component determines spontaneous expressions of emotion. Furthermore, this is the first study to accumulate these findings by empirically testing the typical facial expressions that appear during the experience of intense emotions elicited by films.

### The Upper Parts of Facial Expressions: AU 1, AU 5, and AU 7

We provided three AUs for the upper parts of facial expressions that are related to a number of emotional experiences: inner brow raiser (AU 1; **Figure 3A**), upper lid raiser (AU 5; **Figures 4G,H**) and lid tightener (AU 7; **Figures 3B, 4F**).

The surprise rating positively predicted the occurrence of the inner brow raiser (**Figure 3A**). In other words, AU 1 was found to be more likely to occur when surprise was experienced with greater intensity. Studies of fear expressions are consistent with this combination of the inner brow raiser in the mechanism of eye widening (Susskind and Anderson, 2008). The emergence of the inner brow raiser might be tied to the evolutionary function of cognitive evaluation when locating the source of an unseen startling event (Susskind et al., 2008). Furthermore, this interpretation for the inner brow raiser accords with surprised facial dynamics rooted in the perceptual expectation of observers (Jack et al., 2014). This study seems to empirically reinforce the function of AU 1. Experiences of surprise might be related to other AUs like the outer brow raiser (AU 2) and the upper lid raiser (AU 5), because these facial actions also contribute to the adaptive function of widening the eyes to perceive a greater field of visual information (Ekman et al., 2002). However, Reisenzein et al. (2006) pointed out that it is rare for these AUs to occur simultaneously when people are actually surprised. Thus, as the occurrences of AU 2 and AU 5 were also not predicted by actually surprised experiences in our study, this suggests that the inner brow raiser (AU 1) might be more important in the appraisal of novelty than the outer brow raiser (AU 2) or the upper lip raiser (AU 5).

The upper lid raiser (AU 5) predicted not only experiences of fear, but also experiences of tension (**Figures 4F,G**). Therefore, this facial action might be able to infer people’s tension associated with fear. Fear expressions in BET have been shown to be composed of multiple facial components: inner brow raiser (AU 1), outer brow raiser (AU 2), brow lowerer (AU 4) and upper lid raiser (AU 5, Susskind et al., 2008; Reisenzein et al., 2013). Jack et al. (2014) also showed that the upper lid raiser is a component involved in confusion about the common transmission and expectation of face signals related to surprise and fear. However, our results indicate that the upper lid raiser may contribute to the ability to distinguish surprise from fear, and provide new evidence that the occurrence of AU 5 may implicate a sense of urgency.

Our component-based approach revealed a number of possible relationships that map the lid tightener (AU 7) onto emotional experiences of happiness, contempt, and the absence of relief (**Figures 3B, 4F**). Although many studies regarded the cheek raiser (AU 6) as important components of genuinely happy facial expressions (Frank and Ekman, 1993), our results showed that the adjacent facial activity (i.e., the lid tightener) could be more related to happy experiences than AU 6 as significant results for it were not observed. Mattson et al. (2013) suggested that eye-constriction units such as AU 7 have a systematic association with intense negative and positive emotions. Messinger (2002) also showed in studies of infants that the eye constriction cause by happiness may also be caused by distress. Our results found that the nature of distress caused by AU 7 might be comprised of a lack of relief, considering prior studies of infants. Moreover, a relationship between contempt and the lid tightener (AU 7) is supported by the bidirectional models of the current study. As contempt in the short-term context has been characterized by a kind of derogation toward others (Fischer and Roseman, 2007), AU 7 may be involved in processes where observers perceive humans who are engaging in behavior that is contrary to their standards. Also, previous studies have indicated that eye constriction induced by AU 7 may function to narrow visual sensory stimulation (Susskind et al., 2008). Our findings also suggest that the spontaneous occurrence of AU 7 during an artificially generated moment of piqued distress could be related in some way to the unconscious evolutionary function of the eyelids to constrict and maintain an attention to plausibly distressing stimuli in the external environment. In sum, the empirical findings of the current study attest that AU 7 is involved in emotional experiences as diverse as happiness, relief, and contempt.

### The Lower Parts of Facial Expressions: AU 10, AU 12, and AU 23

In the lower parts of facial expressions, the occurrences of three AUs were significantly correlated with emotional rating values: the upper lip raiser (AU 10; **Figures 3C, 4B,D,F**), the lip corner puller (AU 12; **Figures 4A–F,H**) and the lip tightener (AU 23; **Figure 4I**).

Our proposed bidirectional model provides empirical support that the upper lip raiser (AU 10) is related to the rating values for the actual emotional experience of disgust (**Figures 3C, 4D**). The contempt and unsatisfaction ratings were also positively predicted by the occurrence of this facial component (**Figures 4B,F**). The correlation between disgust and contempt in the current study was also very high (*r* = 0.83). From the perspective of the social functionalist model, disgust and contempt are known to overlap with each other (Hutcherson and Gross, 2011). However, only disgust rating values related to the upper lip raiser (AU 10) were supported by the bidirectional model using model selection methods, not contempt rating values. Thus, disgust might be the more relevant appraisal domain for AU 10 than contempt. This is consistent with the role of upper lip raiser (AU 10) as the characteristic component of disgust in BET (Ekman et al., 2002). Furthermore, Ekman et al. (2002) also suggested that the nose wrinkler (AU 9) may have the same function as AU 10 in prototypical facial patterns of disgust. The current study shows that the upper lip raiser might be more important than the nose wrinkler as a facial reaction in actually felt experiences of disgust. Indeed, Gosselin et al. (2010) showed that 75% of adult participants could express the nose wrinkler as a posed expression of disgust, while only 45% could express the posed expression of the upper lip raiser. These facial actions serve the evolutionary function of avoiding disease from toxic or contaminated food by means of a negative emotional response to something revolting (Rozin et al., 2008). It is plausible that AU 10 is more frequently expressed in spontaneous expressions of disgust while AU 9 is more frequently expressed in posed ones as a function of expression intensity.

The occurrence of the lip corner puller (AU 12) positively predicted the rating values of amusement and contentment in the absence of other people (**Figures 4A,B**). On the other hand, this facial action negatively predicted pain, disgust, embarrassment, contempt, and tension (**Figures 4C–F,H**). The occurrences of the lip corner puller not only predicted positive emotional experiences, but also negative emotional experiences in the opposite direction. Previous research has concluded that smiling is not a reliable marker of positive emotions as it can suppress or conceal negative emotional experiences (Zaki et al., 2009). Rinn (1984) suggested that humans can control the motor actions of the lower face more than those of the upper face, leading to the phenomenon of smile manipulation. This facial action could occur in many contexts like deception (McLellan et al., 2010), relief (Kraft and Pressman, 2012) and greeting (Eibl-Eibesfeldt, 1972). However, the current study revealed that if people are not interacting or sharing the same space with others, the components of the lip corner pullers can be reliably predictors for the existence of positive emotional experiences, and the absence of negative emotional experiences. Therefore, the presence of others appears to influence the occurrence of the facial components involved in smiling. Moreover, considering the findings of the lid tightener (AU 7) related to happiness, the lip corner puller (AU 12) did not include any significant relationship with rating values of happiness. This study provides primarily evidence that distinct positive emotional experiences can map onto specific facial actions.

The spontaneous occurrence of the lip tightener (AU 23) predicted sad experiences (**Figure 4I**). The coherence between actual sadness and AU 23 was replicated under the non-social experimental conditions of watching films to elicit emotion, as well as re-experiencing autobiographical events to induce sadness (Namba et al., 2017). The findings in this study also accord with those described in Namba et al. (2017) which concluded that lip tightening might signify the suppression of sad experiences. Our study extends the cumulative evidence of spontaneous facial expressions of sadness by establishing the role of facial components related to lip tension.

In this way, these results provide preliminary experimental evidence for component-based approaches to emotion-expression coherence, as well as accounts of specific emotions functioning as useful adaptations (Lench et al., 2016). In sum, our results primarily showed that numerous individual facial components mapped onto actual emotional experiences elicited by films in ways that appear to correspond to their evolutionarily functions.

### Limitations and Future Studies

While this study has provided new evidence that there are facial components which underlie a number of actual emotional expressions, there were several limitations. First, when a person experiences individual emotions, the AUs described in this study might appear. However, it would be difficult to consider the expressed AUs in this study as the sole determinant components of individual emotions. Indeed, Scherer et al. (2013) claimed that the observable units of facial behavior are more probabilistic than deterministic. Moreover, the results of the present study measured facial actions in only one context, which was for participants to watch an emotion elicitation film. Hassin et al. (2013) highlighted the role of context when people interpret their internal state through facial expressions. Furthermore, the present study used sixteen emotion rating scales, but the types of emotion elicitation films were limited to the four that encompass surprise, sadness, amusement and disgust. In addition, this experimental scenario also might have affected the relationships between each AU and some of the emotional experiences. Although the participants watched emotion elicitation films in isolation, they could reasonably have concluded that these conditions were created for the sake of the experiment. Thus, to some extent, participating in the experiment itself under these conditions could have created a plausible awareness that the experimental scenario was artificial and thereby affected their display of emotions. Future studies of facial components of emotion should be conducted in multiple contexts, including those in natural settings, or those that can elicit and account for other emotions, and the social emotions.

Also, our result could not make the link between specific facial actions and emotional experiences sufficiently clear because there were crucial temporal gaps between the coded peak facial expressions and the emotional rating values that were measured after watching films. While we applied regression models to fill in these gaps, it must be noted that our results should be interpreted carefully. Despite recent studies denoting the importance of dynamic aspects in facial expressions (Ambadar et al., 2005; Krumhuber and Kappas, 2005), we did not investigate transitions such as appraisal sequences (Scherer et al., 2013), as our analysis was to limited to one rating value per trial. Nevertheless, we have examined the relationships between peak facial expressions and emotional experiences after watching clips based on the argument that the rating values of emotional experiences are capable of anchoring the peak time (Kahneman, 2011). Additional studies should explore the methodologies vetted by previous studies such as real-time physiological arousal, second-by-second continuous ratings of emotional intensity, or cued review of emotions within a time interval to further understand facial components of emotion and overcome this temporal gap (Rosenberg and Ekman, 1994; Mauss et al., 2005; Fernández-dols et al., 2011).

Finally, the results of this study are based on small samples of young Japanese participants. Thus, the regression models requiring hierarchical structures that can include random effects of both participants and stimuli could not converge in this study. Also, we could not explore the asymmetry of facial expressions, which is relevant to the domain of spontaneous facial actions (Mammucari et al., 1988; Ross and Pulusu, 2013; Korb et al., 2016; see review by Murray et al., 2015), because the occurrence rate of applicable action units was too low in our data to evaluate the specificity of sidedness. Moreover, we cannot contribute anything to the debate on cultural differences or the universality of facial components related to emotional experiences. If future studies can collect and corroborate many samples of spontaneous facial components from other populations or remote cultures, it will be very meaningful for facial expression research.

### Summary and Conclusion

This preliminary study used facial components as a basis to provide fine-grained, cumulative, and empirical evidence of new relationships for facial expressions and emotions beyond the prototypical facial patterns promoted by BET. Moreover, we also conducted secret recordings of observable facial expressions to avoid the plausible influence of expressive intentions generated in the presence of others as much as possible. Specifically, we considered each facial component elicited by films as reflecting an actually felt emotional experience on the part of the participant, and our findings suggest that the relationship therein might be related to the underlying adaptive functions of facial expressions. This study took a component-based approach and captured peak facial expressions while people watched film clips designed to elicit target emotions under laboratory circumstances. In addition, while we captured spontaneous reactions as they occur when people are alone by themselves, how these precise components translate to the social sphere remains to be seen. Facial expressions depend heavily on the surrounding context, and it will be necessary to corroborate these findings with data from many other settings going forward.

## Author Contributions

All authors listed, have made substantial, direct and intellectual contribution to the work, and approved it for publication.

## Conflict of Interest Statement

The authors declare that the research was conducted in the absence of any commercial or financial relationships that could be construed as a potential conflict of interest.
